# The complete chloroplast genome sequence of *Ammodendron bifolium* (Fabaceae), an endangered desert shrub from China

**DOI:** 10.1080/23802359.2024.2324922

**Published:** 2024-03-03

**Authors:** Haowen Tian, Xinyu Zhu, Juan Qiu, Hongxiang Zhang, Xiaojun Shi

**Affiliations:** a Xinjiang Key Laboratory for Ecological Adaptation and Evolution of Extreme Environment Biology, College of Life Sciences, Xinjiang Agricultural University, Urumqi, China; b State Key Laboratory of Desert and Oasis Ecology, Key Laboratory of Ecological Safety and Sustainable Development in Arid Lands, Xinjiang Institute of Ecology and Geography, Chinese Academy of Sciences, Urumqi, China

**Keywords:** *Ammodendron bifolium*, chloroplast genome, endangered, fabaceae, phylogenetic analysis

## Abstract

*Ammodendron bifolium*, a rare deciduous shrub, is the only species of *Ammodendron* (Fabaceae) in China, which distributes in Huocheng county, Xinjiang. This study employed high-throughput sequencing technology to assemble the complete chloroplast genome sequence of *A. bifolium*. The entire length of chloroplast genome is 154,426 bp. It comprises 128 genes, which include 85 protein-coding genes, 35 tRNA genes, and 8 rRNA genes. The *A. bifolium* chloroplast genome has a GC content of 36.41%. Phylogenetic analysis strongly supported that *A. bifolium* is sister to the members of the *Sophora* genus. This study will provide the genetic information data for further phylogenetic studies of *Ammodendron*.

## Introduction

**Figure 1.**
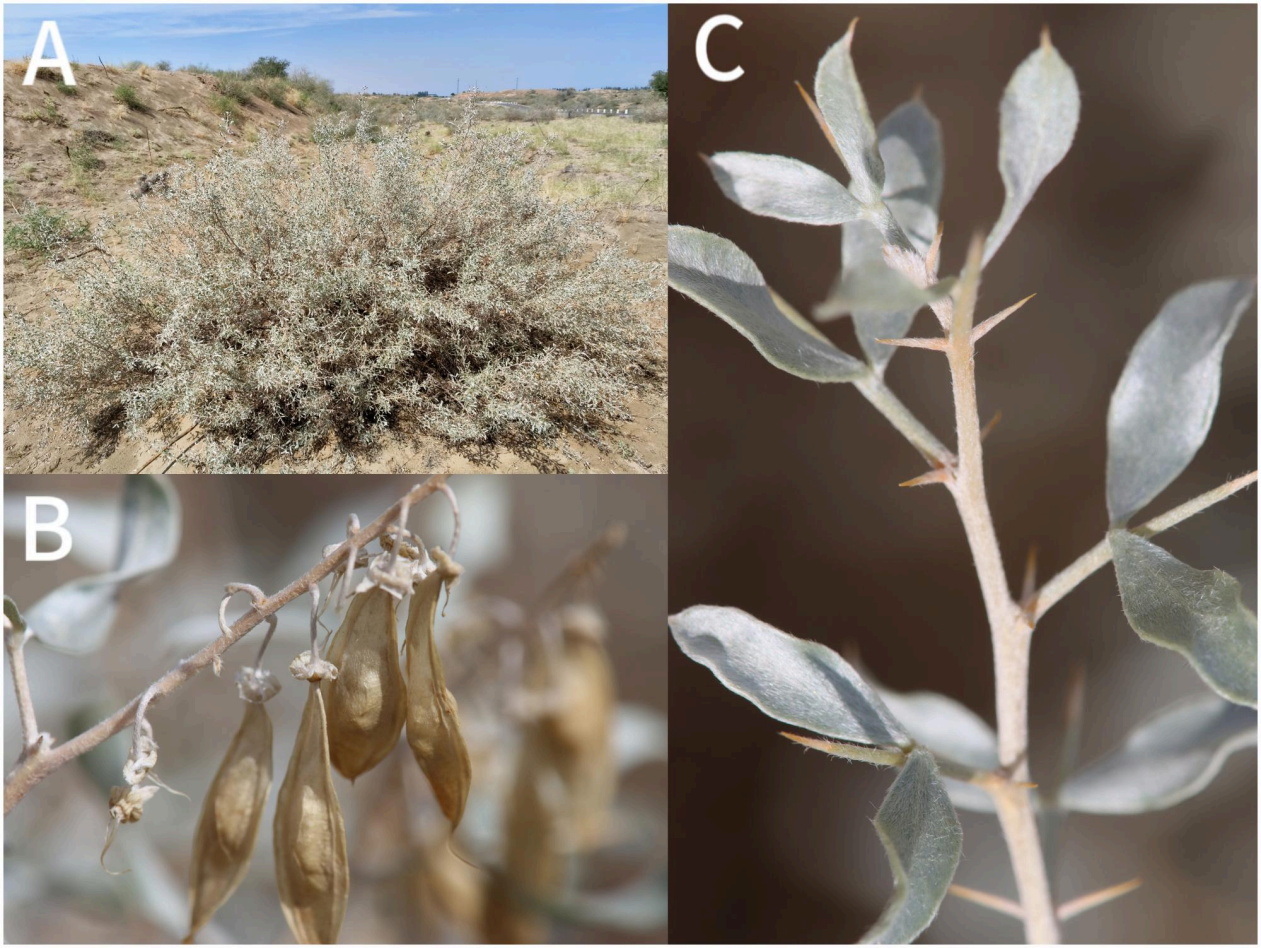
*Ammodendron bifolium*: Vegetative morphology. A. Plant; B. Fruits (pods flat, long, and round lanceolate); C. Stipular thorns (the stipules are thorn-like, the leaflets are arranged in an opposite pattern, and both surfaces of the leaflets are covered with silvery-white sericeous hairs). Photo taken by Xiaojun Shi in Huocheng County.

*Ammodendron bifolium* (Pall.) Yakovlev, 1972, is a rare deciduous shrub and is the only species of *Ammodendron* (Fabaceae) in China (Yakovlev et al. 1996). The distribution of this species is only located in Tukai Desert in Huocheng county, Xinjiang, China. This species usually inhabits extremely arid fixed and semi-fixed deserts. Because of the narrow distribution, habitat destruction and the poor natural renewal ability of *A. bifolium*, it has been classified as a national second-class protected plant in the Information System of Chinese Rare and Endangered Plants (ISCRPE) (http://www.iplant.cn/rep/prot/Ammodendronbifolium). Meanwhile, due to its drought resistance, it can play an important ecological role as a windbreak and sand-fixation plant (Li et al. 2013a, 2013b) (Figure 1). However, the chloroplast genome of *A. bifolium* has not been published yet. In this study, we generated the complete chloroplast genome sequence of *A. bifolium* based on the Illumina paired-end sequencing data, which would be beneficial for further protection and phylogenetic studies on this species.

## Materials and methods

Fresh leaves of *A. bifolium* were collected from the Tukai Desert in Huocheng, Xinjiang Province, China (44°1′N, 80°44′E), and immediately dried using silica gel. The voucher specimen (No. SXJ160) is preserved in the Herbarium of Xinjiang Agricultural University (Contact: Xiaojun Shi, sxj61506@163.com). The collection of plant specimens complies with the Regulations on the Protection of Wild Plants of the People’s Republic of China. Following DNA extraction (Doyle and Doyle 1987), genome sequencing was conducted on the Illumina HiSeq Platform at Genepioneer Biotechnologies Inc, Nanjing, China. The sequencing yielded approximately 7.63 GB of clean data with an average sequencing depth of 2178.71× (Figure S1). The trimmed reads were mainly assembled using SPAdes (Bankevich et al. 2012). Subsequently, the assembled genome was annotated using CpGAVAS (Lohse et al. 2007) and visualized *via* OGDRAW (Liu et al. 2012). CPGview (Liu et al. 2023) was employed to generate cis-spliced and trans-spliced genes, as illustrated in Figures S2 and S3.

**Figure 2.**
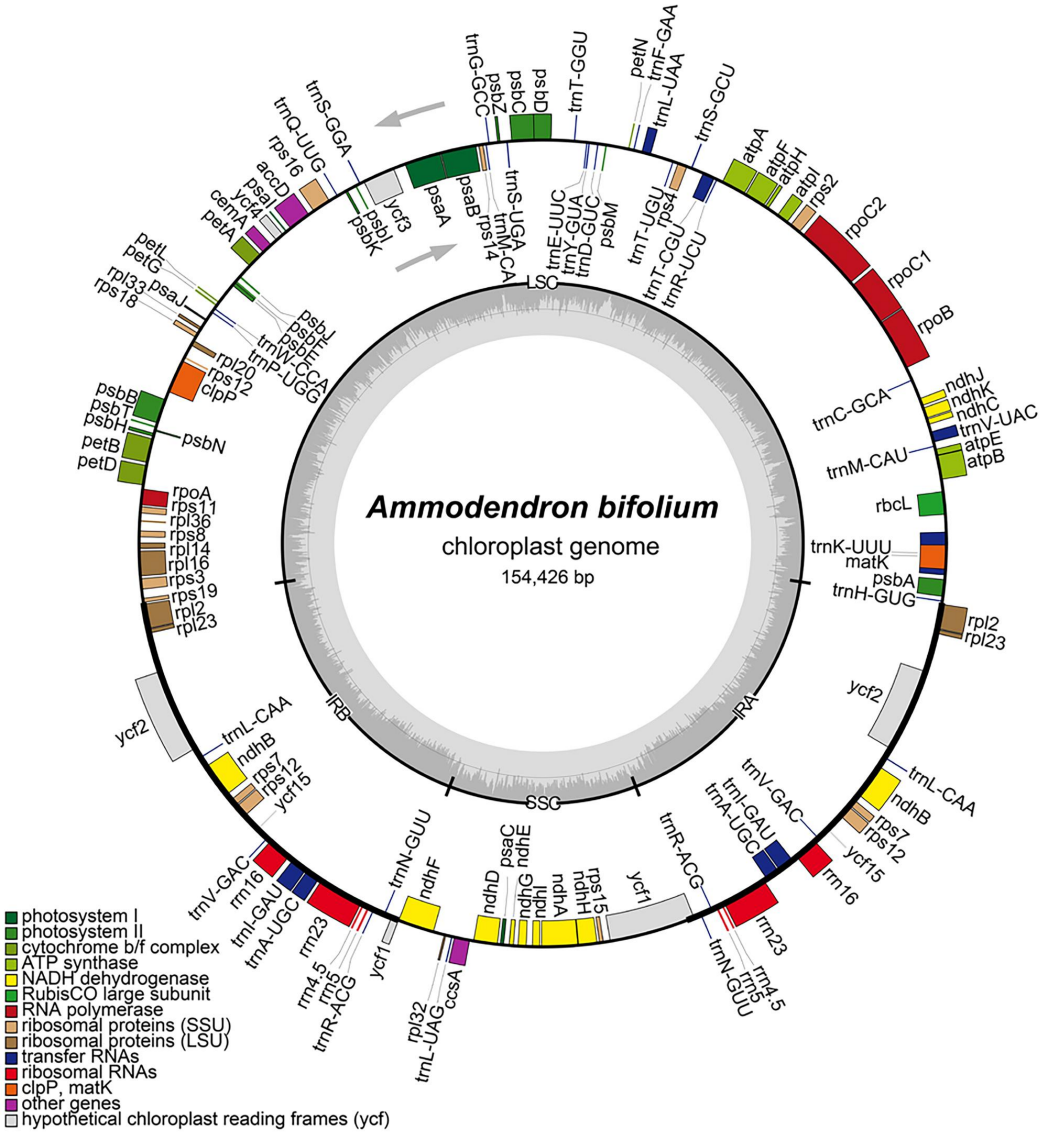
Chloroplast genome maps of *Ammodendron bifolium*. Genes situated on the inner side of the circle undergo transcription in a clockwise direction, while those on the outer side are transcribed in a counterclockwise direction. The inner circle, represented in dark gray, corresponds to the GC content, whereas the light gray denotes the content. Diverse colors symbolize different functional genes. The pronounced line on the large circle delineates the boundaries of the inverted repeat regions (IRa and IRb), segregating the genome into small single-copy (SSC) and large single-copy (LSC) regions.

**Figure 3.**
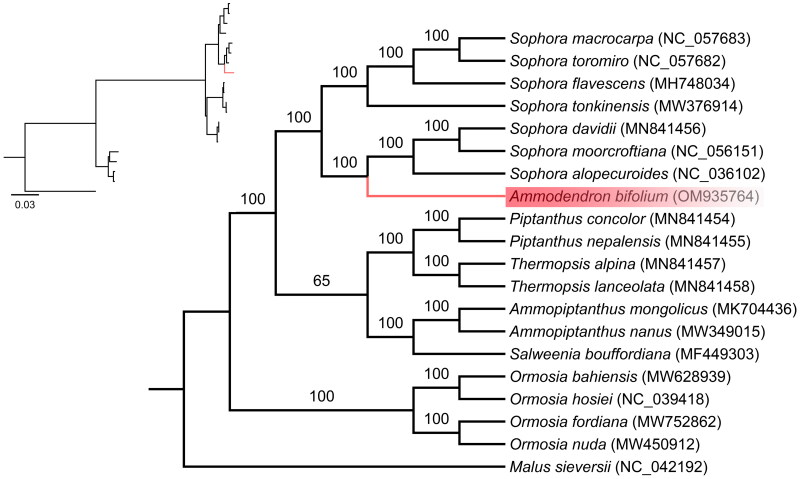
A Phylogenetic tree was reconstructed using the maximum likelihood (ML) method based on the complete chloroplast genome sequences of *Ammodendron bifolium* (shown in red) along with 18 other related species from the Fabaceae family and one species from the Rosaceae family. The numbers on the nodes represent bootstrap values, derived from 1000 replicates. The following sequences were used: *Malus sieversii* (Accession number: NC_042192), *Ormosia nuda* (Accession number: MW450912), *Ormosia fordiana* (Accession number: MW752862), *Ormosia hosiei* (Accession number: NC_039418), *Ormosia bahiensis* (Accession number: MW628939), *Salweenia bouffordiana* (Accession number: MF449303), *Ammopiptanthus nanus* (Accession number: MW349015), *Ammopiptanthus mongolicus* (Accession number: MK704436), *Thermopsis lanceolata* (Accession number: MN841458), *Thermopsis alpina* (Accession number: MN841457), *Piptanthus nepalensis* (Accession number: MN841455), *Piptanthus concolor* (Accession number: MN841454), *Sophora alopecuroides* (Accession number: NC_036102), *Sophora moorcroftiana* (Accession number: NC_056151), *Sophora davidii* (Accession number: MN841456), *Sophora tonkinensis* (Accession number: MW376914), *Sophora flavescens* (Accession number: MH748034), *Sophora toromiro* (Accession number: NC_057682), *Sophora macrocarpa* (Accession number: NC_057683).

To determine the phylogenetic position of *A. bifolium* within the Fabaceae family, we obtained 19 complete chloroplast genomes of Fabaceae species from GenBank and used *Malus sieversii*, a member of the *Malus* genus in the Rosaceae family as the out-group. We aligned the complete chloroplast genome sequences of *A. bifolium* and the other 19 species using MAFFT (Katoh and Standley 2013) and performed phylogenetic analysis based on maximum likelihood using IQ-TREE 1.6.8 (Nguyen et al. 2015) with the TVM + F + I + G4 nucleotide substitution model, which was selected by ModelFinder (Kalyaanamoorthy et al. 2017).

## Results and discussion

The whole chloroplast genome of *A. bifolium* (accession number: OM935764) has a length of 154, 426 bp and comprises a large single-copy region (LSC, 84, 486 bp), a small single-copy region (SSC, 17, 946 bp) and two inverted repeats (IRs, 25, 997 bp each) (Figure 2). The LSC, SSC, and IR regions have GC contents of 33.91%, 30.07%, and 42.66%, respectively, resulting in an overall GC content of 36.41% for the *A. bifolium* chloroplast genome. The genome contains 128 functional genes, of which 85 are protein-coding genes, 35 are tRNA genes, and 8 are rRNA genes. Each of thirteen protein-coding genes (*rpo*C1, *atp*F, *rps*16, two *rps*12, *pet*B, *pet*D, *rpl*16, two *rpl*2, two *ndh*B, *ndh*A) and seven tRNA genes (*trn*K-UUU, *trn*V-UAC, *trn*T-CGU, *trn*L-UAA, two *trn*I-GAU, *trn*A-UGC) have one intron, while two protein-coding genes (*ycf*3 and *clp*P) have two introns.

To investigate the phylogenetic relationship between *A. bifolium* and its related taxa, we obtained the complete chloroplast genomes of 19 other closely related species from the NCBI GenBank database. Subsequently, a maximum likelihood (ML) analysis was performed to construct a phylogenetic tree (Figure 3). Phylogenetic analysis revealed that *A. bifolium* was clustered with *Sophora alopecuroides*, *Sophora moorcroftiana* and *Sophora davidii*, with *Ammodendron* being nested within the *Sophora* clade. These findings provide support for the non-monophyletic nature of the *Sophora* genus, which is consistent with the previously research about the phylogenetic relationships among genera in Fabaceae, as determined by using both nrDNA and plastid DNA regions (Duan et al. 2019). Further research is required to determine the phylogenetic relationship between *Ammodendron* and *Sophora*.

## Conclusions

The complete chloroplast genome of *Ammodendron bifolium* reported for the first time in our study. It had a typical circular structure composed of 154,426 bp and 128 genes. Based on molecular phylogenetic analysis using 20 complete chloroplast genome, we provided evidence that the *Sophora* genus were the non-monophyletic nature, as *A. bifolium* was closely related to *Sophora* in the tribe *Sophoreae* and nested within the *Sophora* clade. The published chloroplast genome of *A.bifolium* will provide the genetic information data for further phylogenetic studies of *Ammodendron*.

## Ethical approval

The data collection was conducted in accordance with the policies of the International Union for Conservation of Nature (IUCN) pertaining to research on species at risk of extinction (refer to the Guidelines for appropriate uses of IUCN Red List data). Additionally, the Convention on Biological Diversity and the Convention on the Trade in Endangered Species of Wild Fauna and Flora were also adhered to.

## Supplementary Material

Supplemental Material

## Data Availability

The data that support the findings of this study are openly available in GenBank of NCBI at https://www.ncbi.nlm.nih.gov, reference number OM935764. The associated BioProject, SRA, and Bio-Sample numbers are PRJNA845065, SRR19537695, and SAMN28854403, respectively.
