# Supplementary material for: The complete chloroplast genome sequence of Ammodendron bifolium (Fabaceae), an endangered desert shrub from China
Source: Mitochondrial DNA B Resour. 2024 Mar 3;9(3):309–13. doi: 10.1080/23802359.2024.2324922 (PMC10913713; doi:10.1080/23802359.2024.2324922)
Supplement: Supplemental Material [file TMDN_A_2324922_SM8908.docx]

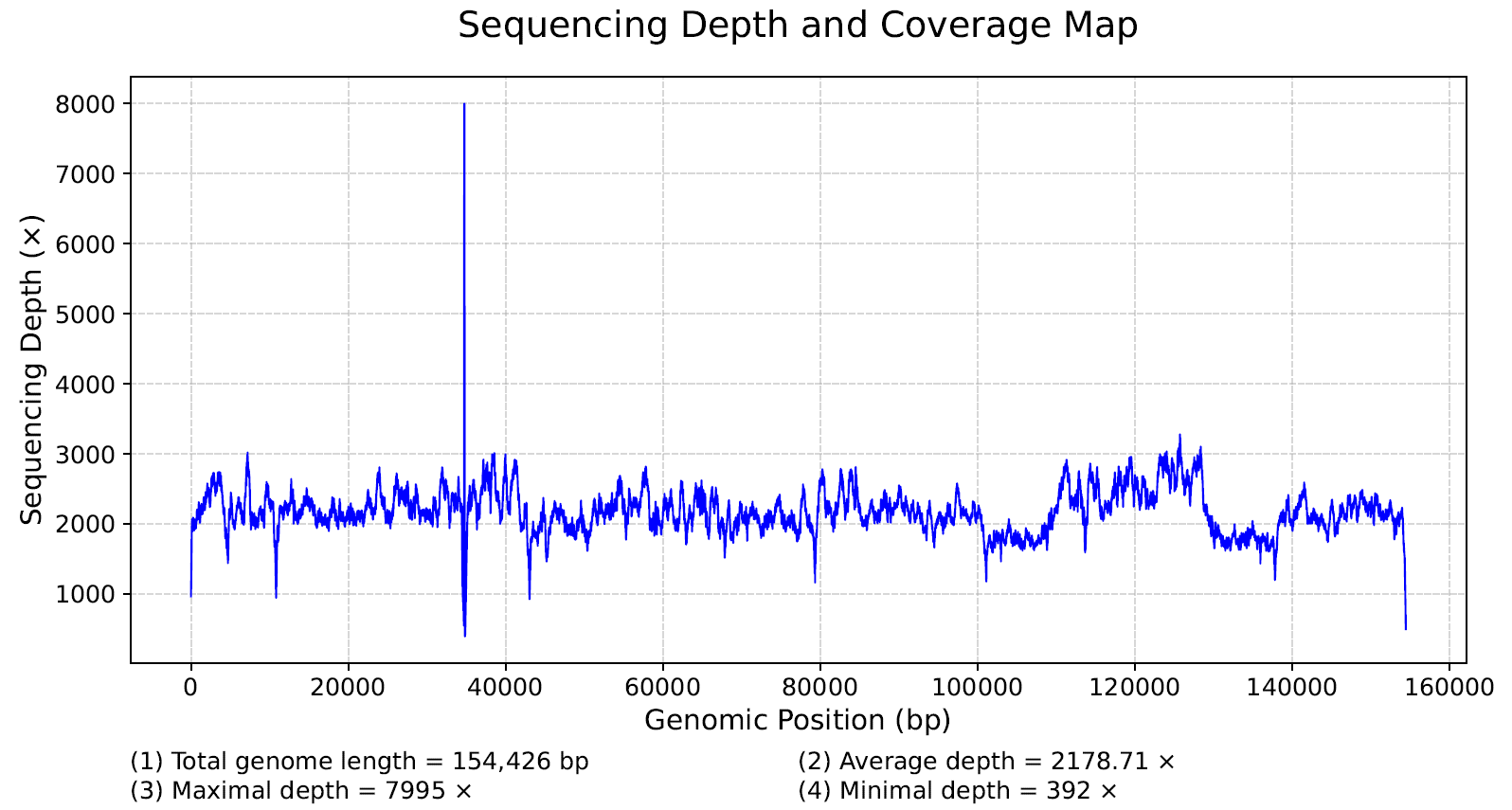


Figure S1 Coverage depth figure of the *Ammodendron bifolium* chloroplast genome. The horizontal coordinate is the position of the chloroplast genome, and the vertical coordinate is the sequencing depth.


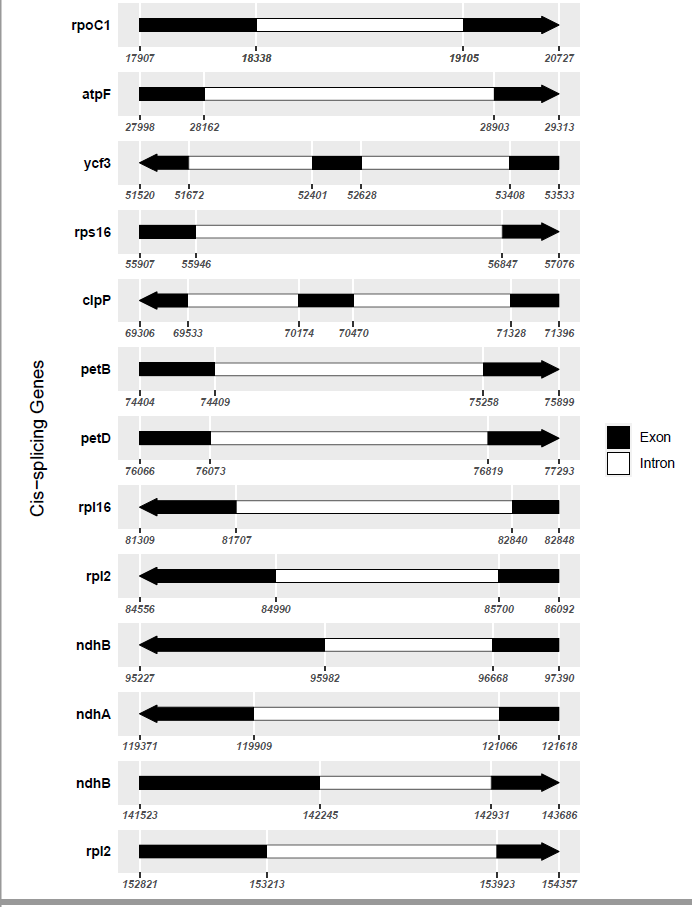


Figure S2 Schematic map of the cis-splicing genes in *Ammodendron bifolium* chloroplast genome.


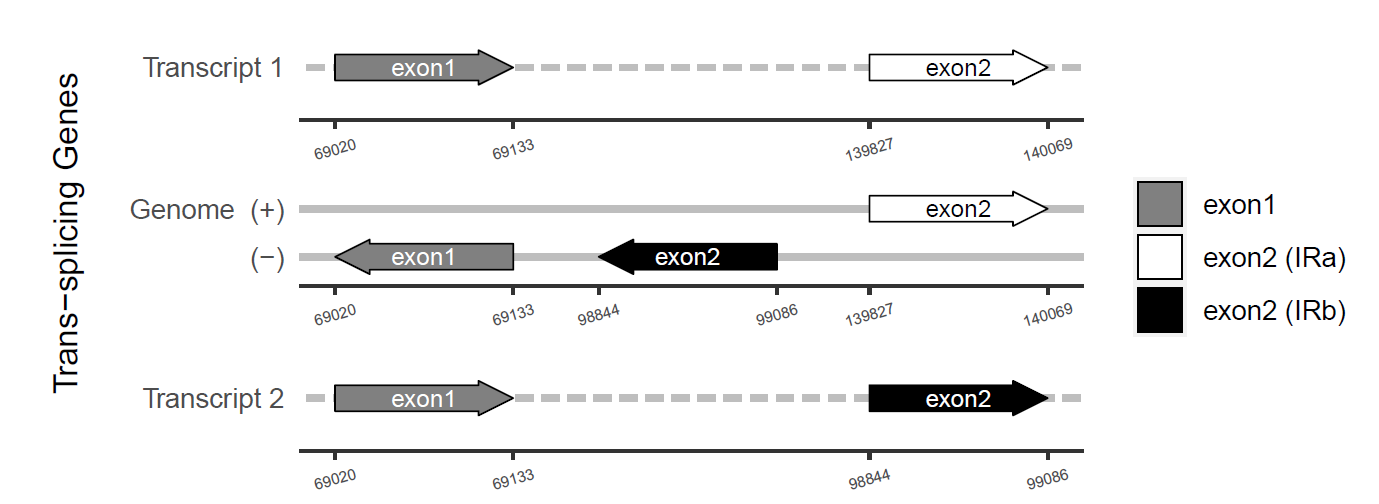


Figure S3 Schematic map of the trans-splicing gene in *Ammodendron bifolium* chloroplast genome.
